# Case Report: Simple Triple of the Median Maxillary Labial Frenum

**DOI:** 10.1155/crid/9929298

**Published:** 2026-07-16

**Authors:** Hatim Mohammed Almahdi, Hussain Adel Alghafli, Abdullah Othman Alasafirah, Mousa Haney AlSaleem, Mohammed Al Amer, Hussain Mohammed Alkhames

**Affiliations:** ^1^ Oral and Maxillofacial Surgery and Diagnostic Sciences Department, College of Dentistry, King Faisal University, Al Ahsaa, Saudi Arabia, kfu.edu.sa; ^2^ College of Dentistry, King Faisal University, Al Ahsaa, Saudi Arabia, kfu.edu.sa

**Keywords:** case report, frenum, labial frenum, median maxillary labial frenum

## Abstract

The median maxillary labial frenum (MMLF) is an anatomical structure composed of mucous membrane and connective tissue within the oral cavity. It serves as a key component of the septo‐premaxillary traction system, extending from the nasal septum to the mucosal aspect of the lip and from the midline of the lip anteriorly to the median interincisal suture. The frenum exhibits dynamic and variable morphology, with its shape, size, and position changing throughout various stages of growth and development. A 20‐year‐old Saudi male presented to the King Faisal University Dental Complex Clinics. The patient′s medical, dental, and family histories were unremarkable, with no syndromic conditions reported among family members. Both extraoral and intraoral examinations were within normal limits, except for the presence of a simple triple labial frenum. Direct visual inspection was performed to evaluate the frenum′s form, size, and point of attachment. Blanching or tension was assessed by gently drawing the upper lip outward and upward. The simple triple frenum type of MMLF is a rare anatomical variation, with a reported prevalence ranging from 1.0% to 1.57%. The frenum is classified as pathogenic when associated with midline diastema, gingival recession, loss of papilla, interdental bone loss, reduced lip mobility, difficulty with oral hygiene, and malalignment of teeth. Pathogenic frena may also present with a flattened papilla closely attached to the gingival margin, insufficiently attached gingiva, and a shallow vestibule. Emphasizing frenum assessment during oral examinations is essential to prevent misdiagnosis of normal anatomical variations as pathological findings. Accurate identification supports appropriate intervention and effective treatment planning.

## 1. Introduction

The frenum is a fold of mucous membrane connecting the lip to the alveolar process at the midline of the maxilla and mandible. It originates from central vestibular lamina cells and is primarily composed of connective tissue and epithelium, occasionally containing muscle fibers [1, 2].

The frenulum labii superioris, or median maxillary labial frenum (MMLF), is an anatomical structure formed by a mucous membrane and connective tissue in the oral cavity. It can be defined as a “fibrous band of tissue attached to the bones of the mandible and maxillae and is present superficial to muscle attachments” [3]. It has a wide and relatively deep origin or base on the inner surface of the upper lip and extends to the middle portion of the buccal surface of the alveolar process between the central incisors.

The frenum is a dynamic and variable structure, subject to changes in shape, size, and position during different stages of growth and development. During growth, it tends to diminish in size, evolving from wide and thick to thinner and smaller [4].

A normal frenum attaches to the free gingival margin in a way that does not exert a pull on the zone of the attached gingiva. It usually terminates at the mucogingival junction. However, its level may vary from the vestibule height to the crest of the alveolar ridge and even to the incisal papilla area in the anterior maxilla.

Several approaches have been presented in the literature to categorize the various types of maxillary midline frena based on morphology, the most commonly used being described by Sewerin and Gumusboga [5, 6].

The most common types of MMLF observed were the simple single frenum (63.79%) and the simple triple frenum (1.57%) [7]. No statistically significant difference was found between males and females in these variations. However, there are rare reports available in the literature concerning these variations.

This case is presented to inform dental practitioners about the potential occurrence of this anomaly. Accurate diagnosis of a malformed labial frenum is necessary to guide optimal intervention, highlighting the importance of careful assessment of the maxillary frenum during intraoral examination. This report provides baseline data for future research on the rare simple triple maxillary labial frenum.

## 2. Case Presentation

A 20‐year‐old Saudi male presented to the King Faisal University Dental Complex Clinics, Al Ahsa, Saudi Arabia, for a routine dental check‐up. The patient′s medical history was unremarkable, with no known allergies or syndromes. His dental history was notable for multiple restorations, while his family history was insignificant.

### 2.1. Clinical Examination Method

**Figure 1:**
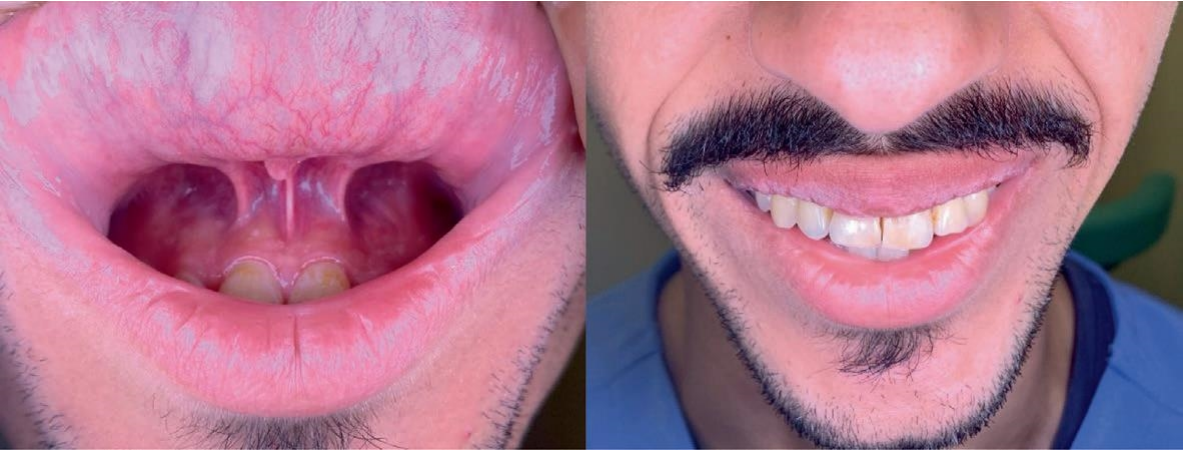
Simple triple of the median maxillary labial frenum.

Both extraoral and intraoral examinations were within normal limits, except for the presence of a simple triple labial frenum (Figure 1). Patient preparation and positioning: The patient is seated in a dental chair with suitable lighting. The main investigator carefully lifts the upper lip upward and outward, away from the alveolar process, using the index fingers and thumbs of both hands to reveal the labial vestibule and the frenal connection. Direct visual inspection: Observe the frenum′s form, size, and point of attachment. Next, assess for blanching or tension by gently drawing the upper lip outward and upward. Movement of the interdental papilla or blanching (whitening) of the papilla tip during this maneuver indicates a positive finding—suggesting an aberrant or pathological frenum [8]. None of the family members had any syndromes. Written informed consent was obtained from the patient for the publication of this case report and any accompanying images according to the Research Ethics Committee at King Faisal University protocol (KFU‐REC‐2024‐OCT‐ETHICS2530).

## 3. Discussion

The MMLF is a mucosal fold that attaches the lip to the alveolar mucosa, gingiva, and periosteum, limiting movement. Various morphological variations and types of frenal attachments are observed in primary, mixed, and permanent dentition (Table 1). Its composition consists of epithelium, collagen fibers, blood vessels, nerves, and occasionally, a few elements of minor salivary glands and isolated striated muscle fibers [2].

**Table 1:** Summary of the main characteristics of the included studies.

No.	Author, year	Title	Study design	Country
1	Joshi et al., 2021 [9]	Variations in Maxillary Frenal Morphology in a Sample of Newari Children of Bhaktapur	Cross‐sectional	Nepal
2	Rijal et al., 2024 [10]	Prevalence of Maxillary and Mandibular Frenal Attachment and Its Association With Age, Gender, and Oral Hygiene Status in Nepalese Population Seeking Dental Treatment	Cross‐sectional	Nepal
3	Zakirulla et al., 2021 [11]	Maxillary Midline Frenum Morphology and Its Variations in Saudi Children	Cross‐sectional	Saudi Arabia
4	Gujjari et al., 2012 [12]	Frenectomy: A Review With the Reports of Surgical Techniques	Review	
5	Rajani et al., 2018 [13]	Prevalence of Variations in Morphology and Attachment of Maxillary Labial Frenum in Various Skeletal Patterns ‐ A Cross‐Sectional Study	Cross‐sectional	India
6	Madi et al., 2024 [14]	Associations Between Maxillary Labial Frenum Morphology, Attachment, and Patient‐Related Clinical Factors in Saudi Arabian Adults	Cross‐sectional	Saudi Arabia
7	Biradar et al., 2020 [2]	Assessment of Diverse Frenal Morphology in Primary, Mixed, and Permanent Dentition: A Prevalence Study	Cross‐sectional	

The MMLF is an essential constituent of the septo‐premaxillary traction system that extends from the nasal septum to the mucosal part of the lip and from the middle of the lip in front to the median interincisal suture. The activity of the nasolabial muscles is thus transmitted to this system, regulating symmetrical facial growth and providing stability to the upper lip. Therefore, it is crucial to preserve the integrity of the maxillary labial frenum during surgical procedures involving the nasal septum, especially in cases of cleft lip and palate [15].

The MMLF stabilizes the upper lip, representing the connection between the upper lip′s tubercle and the palatine papilla [7]. Initially, the frenal attachment is higher in the middle of the attached gingiva, but as development progresses, it shifts more apically, below the mucogingival junction.

The most common type of MMLF is the simple single frenum, which accounts for more than 60% of cases in studies conducted across different populations [5, 7, 10]. In contrast, the prevalence of the simple triple frenum type of MMLF is rare, ranging from 1.57% among Indian populations, as reported by Mohan et al. and Madi et al., to 1.0% in Saudi populations [7, 14].

Diverse variations in MMLF morphology may occur, which are considered developmental rather than pathological and have been identified across all age groups [16]. However, the presence of abnormal frena has been reported in the literature as a feature of syndromic conditions such as Ehlers–Danlos syndrome, infantile hypertrophic pyloric stenosis, holoprosencephaly, Ellis–van Creveld syndrome, and orofacial digital syndrome [2, 17, 18]. Each syndrome exhibits relatively specific frenal abnormalities, ranging from multiple, hyperplastic, or hypoplastic frena to the absence of frena.

The maxillary midline diastema, located between the maxillary central incisors, is relatively common during mixed dentition. It is often due to the collagen fibers of the labial frenum disrupting the transseptal fibers of the periodontal ligament, typically found between the central incisors. This disruption is associated with the midline diastema, and the failure of the frenum to migrate apically during development has been identified as a causative factor in its persistence. It may also be caused by the insertion of the labial frenum into a notch in the alveolar bone, resulting in a band of heavy fibrous tissue lying between the central incisors [4].

The gingival recession has been reported with the attachment of certain types of maxillary frena, especially gingival and papillary, at a crestal insertion point close to the gingival margin of the incisors. This can retract the marginal gingiva or papilla, thereby contributing to the initiation or progression of periodontal disease. Furthermore, oral hygiene procedures may become complicated, and the accumulation and retention of plaque can be affected by the anterior frenal attachment in the maxilla [19].

The MMLF is considered a complicating factor in denture construction, with the most frequent denture‐induced lesions occurring at the frena and muscular attachment regions in the maxillary vestibular sulcus [20].

The MMLF can also be a potential cofactor for perimucositis and peri‐implantitis and may cause difficulties in speech, mastication, and esthetics. It may hinder the upper lip from forming a proper seal, making it difficult for children to breastfeed and leading to nutritional deficiencies, difficulties with brushing, and compromised oral hygiene [4, 12, 21].

Injuries to the jaw and face in early childhood may cause MMLF abnormalities, and surgical resection or frenectomy can potentially expose parts of the root surface, leading to gingival recession [13].

Abnormal frena are detected visually by applying tension over the frenum to observe the movement of the papillary tip or the blanching produced due to ischemia in the region. The frenum is characterized as pathogenic when it is unusually wide, when there is no apparent zone of attached gingiva along the midline, or when the interdental papilla shifts upon extension of the frenum. Additionally, a pathogenic frenum can present with a flattened papilla closely attached to the gingival margin, leading to gingival recession and hindrance to oral hygiene. An inadequately attached gingiva and a shallow vestibule may also be observed [7].

The frenum is characterized as pathogenic when it is associated with midline diastema, gingival recession, loss of papilla, interdental bone loss, poor lip mobility, difficulty in brushing, and malalignment of teeth. It is also considered pathogenic when a flattened papilla with the frenum closely attached to the gingival margin is present, along with an inadequately attached gingiva and a shallow vestibule [12]. In cases of pathological MMLF, diagnosis was made as the one associated with midline diastema, initially closed with fixed orthodontic appliances, and frenectomy should be conducted as a second step.

Surgical interventions for pathological frena include various procedures such as *frenectomy* (complete removal), *frenotomy* (simple incision or cutting), and *frenuloplasty* (repositioning of the aberrant muscular attachment). These procedures can be performed using either scalpel‐based or laser‐based techniques, which are procedures often associated with minimal bleeding, reduced need for sutures, faster healing, and decreased postoperative discomfort [22].

Surgical intervention involving the MMLF in children should be deferred until the eruption of the permanent canines and only considered following orthodontic closure of the midline diastema or in conjunction with active orthodontic treatment, as the physiological pressure from the erupting canines may close the diastema [23].

## 4. Conclusion

Frenum assessment during oral examination is crucial to avoid misdiagnosing normal anatomical variations as pathological, thereby supporting appropriate intervention and effective treatment planning. Further research is needed to evaluate the development of the maxillary labial frenum across different age groups and its morphological changes, enabling timely intervention in cases of abnormal development.

## Author Contributions

The authors Hatim Mohammed Almahdi, Hussain Adel Alghafli, Abdullah Othman Alasafirah, Mousa Haney AlSaleem, Mohammed Al Amer, and Hussain Mohammed Alkhames contributed to the study′s conception and design, as well as to data acquisition, analysis, and interpretation. Hatim Mohammed Almahdi wrote the paper, and Hussain Adel Alghafli, Abdullah Othman Alasafirah, Mousa Haney AlSaleem, Mohammed Al Amer, and Hussain Mohammed Alkhames reviewed and edited it.

## Funding

No funding was received for this manuscript.

## Disclosure

All authors have read and approved the final version of the manuscript. Hatim Mohammed Almahdi, the corresponding author, had full access to all data in this study and takes complete responsibility for the integrity and accuracy of the data analysis.

## Ethics Statement

The authors certify that they have obtained all appropriate patient consent forms and institutional forms. The Research Ethics Committee at King Faisal University grants its ethical approval to the protocol (KFU‐REC‐2024‐OCT‐ETHICS2530). The patient gives his consent for the images and other clinical information to be reported in the journal. The patient understands that his name and initials will not be published, due efforts will be made to conceal his identity, and anonymity is guaranteed.

## Conflicts of Interest

The authors declare no conflicts of interest.

## Data Availability

The data that support the findings of this study are available from the corresponding author upon reasonable request.
